# Sacred space in motion: geography, time, and texts in the historiography of Lingyan Temple

**DOI:** 10.3389/fsoc.2026.1720249

**Published:** 2026-04-29

**Authors:** Bin Han, Yaoping Liu

**Affiliations:** Department of Global Buddhism, Institute of Science Innovation and Culture, Rajamangala University of Technology Krungthep, Bangkok, Thailand

**Keywords:** bibliography, Buddhist historiography, Chinese Buddhism, chronology, geography, Lingyan Temple, sacred space

## Abstract

This study examines the historiography of Lingyan Temple, a prominent Buddhist temple in Jinan, through an empirical and positivist lens. The study delineates the temple’s historical evolution from the Eastern Jin dynasty to the present, employing a synthesis of geographical analysis, archaeological findings, epigraphic documentation, and bibliographic references. The analysis emphasizes three interrelated facets of Lingyan’s historiography. The temple’s prominent role in regional Buddhist networks was primarily influenced by its strategic location at the intersection of cultural and political routes in northern China. Second, the historical reconstruction of Lingyan’s beginning, rise, fall, and restoration shows how the development of Buddhist temples goes in cycles, closely tied to changes in dynasties and the way people give money to temples. Third, the bibliographic evaluation of primary sources, including dynastic histories, temple gazetteers, and epigraphic inscriptions, highlights the methodological difficulties and the abundance of recreating temple history experimentally. These studies show that “sacred space” is not a set idea; it is always being rethought by history, geography, and written traditions. The research contends that a positivist methodology in Buddhist temple historiography enables a more systematic, verifiable, and comparable framework for analyzing religious heritage. This methodology enriches global dialog on the documentation, interpretation, and revitalization of religious heritage over time, while also offering insights into broader concerns related to the material, temporal, and textual aspects of sacred space in Chinese Buddhism, especially Lingyan Temple.

## Introduction

For an extended period, the examination of Buddhist temples has been a critical component of comprehending the history of Chinese religion. They were sacred spaces for ritual practice, functioning as centers of learning, political negotiation, and cultural production (Davis, 2020; Wu, 2022). Recent research has underscored the significance of examining temples, such as Lingyan in Jinan, which are renowned for their massive sculptures and inscriptions, in relation to both local and transregional networks of Chinese Buddhism (Cho and Kang, 2019; Gao et al., 2024). Nevertheless, the historiography of these institutions has often been descriptive or narrative, predominantly utilising gazetteers and biographies without rigorous empirical analysis (Huang and Luo, 2021; Chen and Wiles, 2020). Holy space research has dramatically improved our understanding of how religious locations are created and maintained through rituals and social and political factors. Despite this, a significant number of individuals continue to perceive sacred space as a symbolic construct (Ju, 2023; Tang and Wang, 2023). This inclination may result in inadequate recognition of the profound influence of geographical location and temporal cycles on institutional development (Obadia, 2025; Fu et al., 2025). Additionally, whereas dynastic histories, inscriptions, and temple gazetteers offer plentiful resources, their application as empirical evidence occasionally lacks rigorous validation. Consequently, a method that systematically reconstructs temple trajectories via triangulated and empirical history is essential (Kieschnick, 2019; Liu and Tian, 2021).

**Figure 1:**
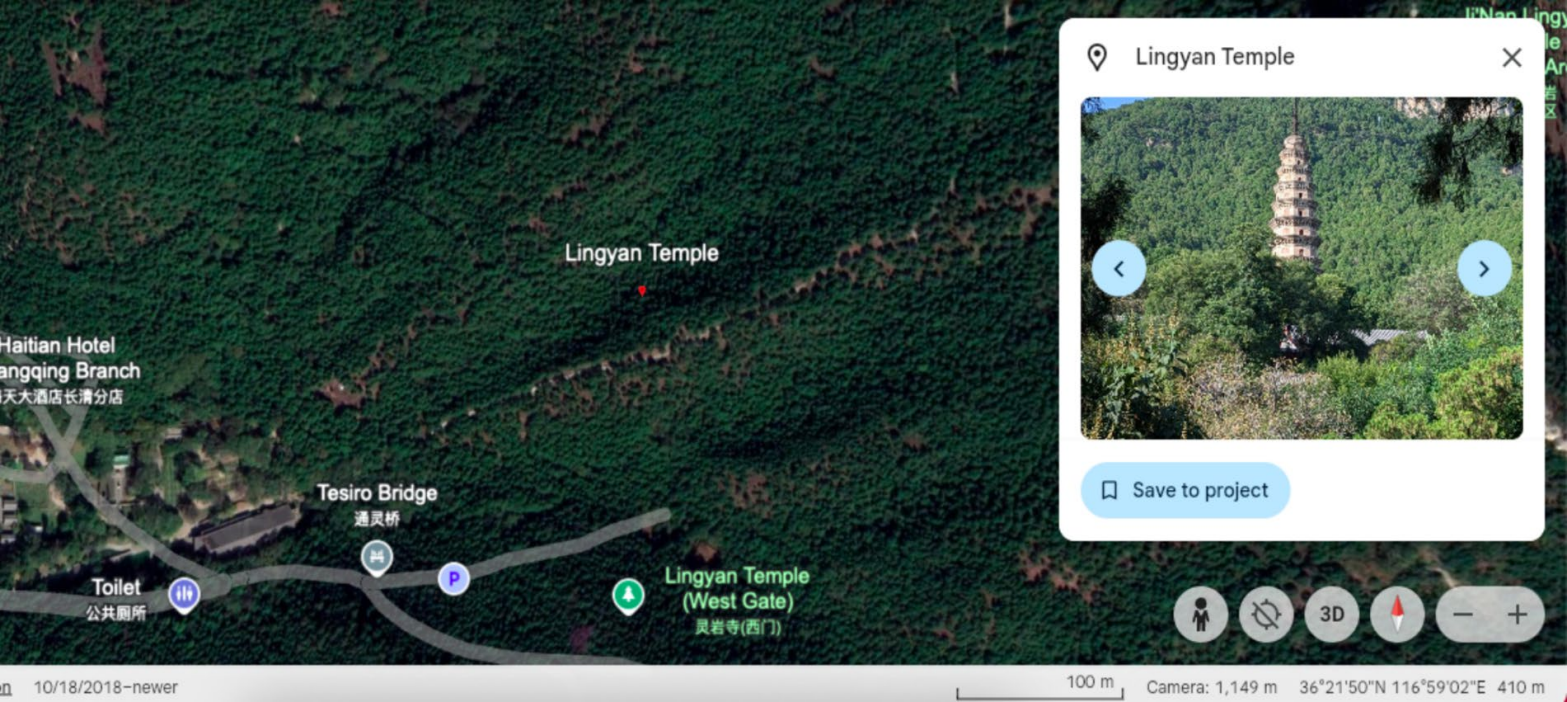
Geographical location of Lingyan Temple.

**Figure 2:**
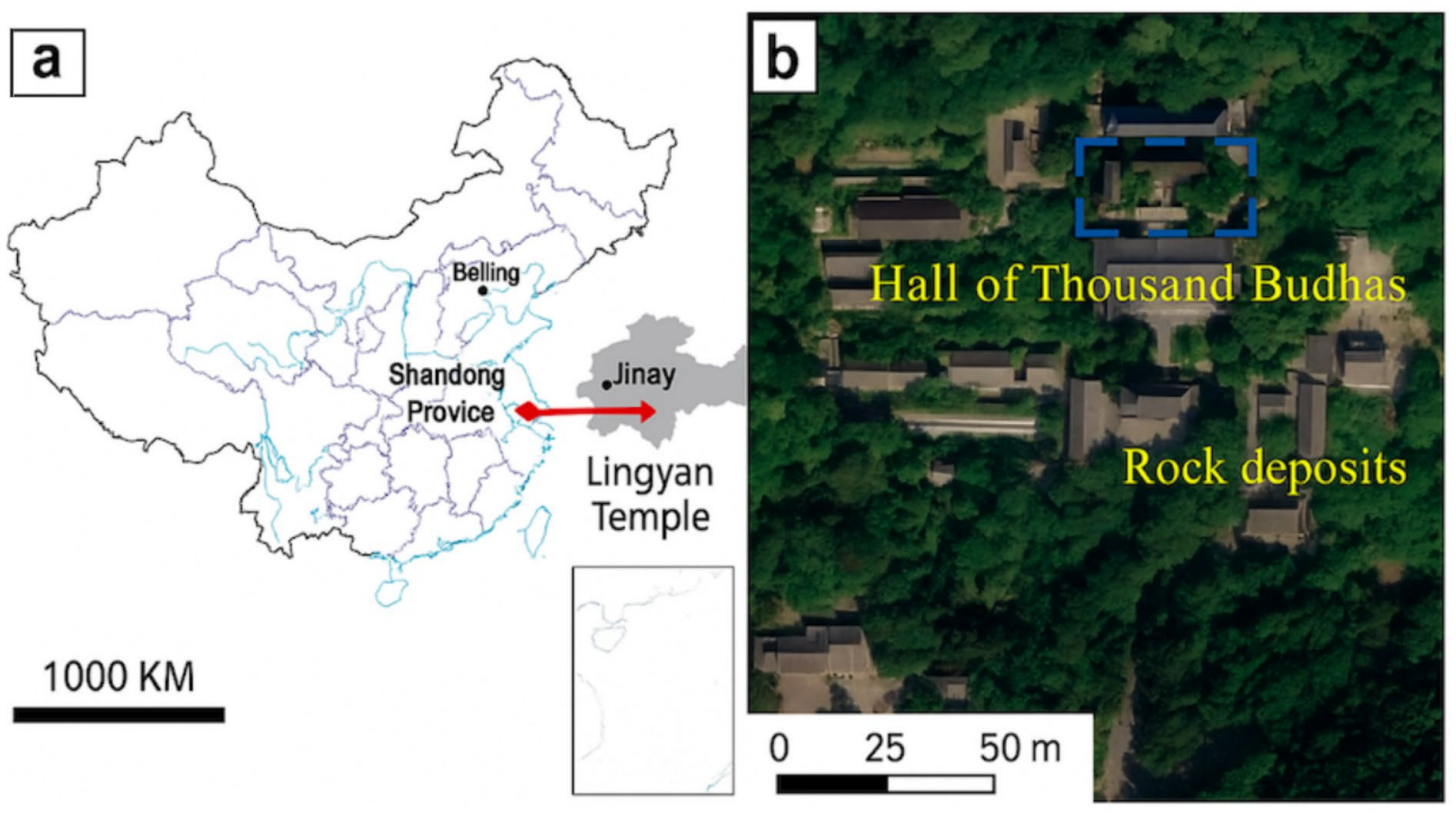
The distribution area of the weathering stone objects in the Lingyan Temple.

**Figure 3:**
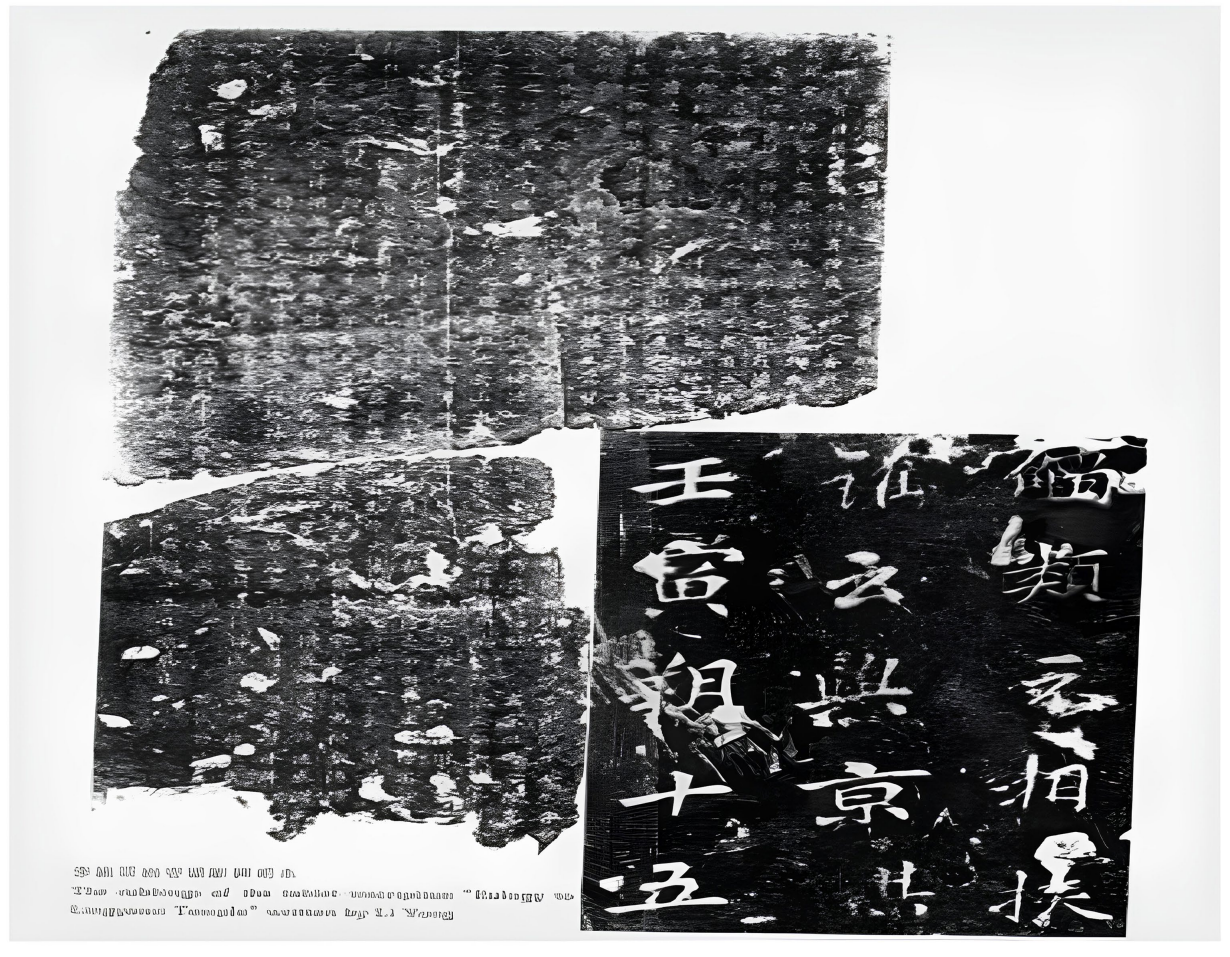
The inscription taken from Lingyan Si.

This study fills these gaps by providing a positivist historiography of Lingyan Temple, concentrating on three interconnected aspects: geography, chronology, and bibliography. The term “historiography” is defined as the reconstruction of institutional history through the verification of geographical, archaeological, and textual evidence, rather than a review of secondary historical writings. First, the geographical context of Lingyan Temple is analyzed concerning trade routes, cultural landscapes, and political hubs in northern China, reflecting contemporary research on the influence of spatial placement on Buddhist institutional resilience (Chen and Wiles, 2020; Huang and Luo, 2021; Fu et al., 2025; Gao et al., 2024). This analysis underscores how location not only contributed to the temple’s ascendance in significance but also strategically situated it within regional Buddhist networks, akin to findings regarding Mount Putuo and Wutai, where geography was pivotal in the expression of sacred authority (Wu, 2022; Obadia, 2025). Second, triangulating archaeological evidence, epigraphic records, and authenticated historical documents to reconstruct Lingyan Temple’s establishment, flourishing, decline, and revitalization aligns with recent efforts to integrate epigraphy and archaeology for verifiable cultural heritage timelines (Liu and Tian, 2021; Fu et al., 2025). This reconstruction confirms comparative studies of temple trajectories during the Tang and Song eras that the temple’s life cycle is similar to other patterns of continuity and break in Chinese Buddhist history (Cho and Kang, 2019; Liu and Tian, 2021). Third, the bibliographic assessment of primary sources, such as dynastic histories, temple gazetteers, and inscriptions, shows their abundance and methodological challenges, such as political bias, mythologization, and retrospective editing, which are increasingly recognized in East Asian Buddhist critical historiography. According to this research, sacred space should be seen as dynamic through multidimensional integration (Figures 1–3).

### Defining the positivist historiographical framework

In this study, positivist historiography refers to a method of reconstructing institutional history through systematic verification of empirical evidence, integrating material, spatial, and textual data to distinguish demonstrable historical events from later interpretive narratives. Following Gill et al. (2018) and Kala (2025), positivist historiography emphasizes three core principles: (1) source triangulation across epigraphic, archaeological, and textual records; (2) critical source evaluation through analysis of ideological and political biases embedded in gazetteers and dynastic histories; and (3) empirical corroboration via stratigraphic, palaeographic, and spatial validation. This framework does not reject interpretative dimensions but situates them after verifiable historical baselines have been established. In Buddhist institutional historiography, this approach aligns with recent methodological advancements by Kieschnick (2019), Huang and Luo (2021), and Davis (2020), who argue that inscriptions and archaeological layers must serve as primary evidence, while textual sources require critical contextualization. Accordingly, the present study employs positivist historiography as a reproducible analytical procedure that meets contemporary standards for reconstructing the historical development of Lingyan Temple.

Research question:

- *RQ1. How do geographical factors influence the historical development and strategic position of Lingyan Temple from the Eastern Jin period to the modern period?*
- *RQ2. How can the chronological structure of the establishment, progress, decline, and revitalization of Lingyan Temple be reconstructed based on archaeological evidence, inscriptions, and verified historical documents?*
- *RQ3. What are the characteristics of the main bibliographic sources in compiling the historical narrative of Lingyan Temple empirically?*

## Literature review

### Geography and the strategic position of Buddhist temples

Geographical factor becomes a critical factor in shaping the historical trajectory and contemporary significance of Buddhist temples in East Asia. Recent scholarship in China has increasingly highlighted that the development of temples is not solely determined by ritual requirements but is also influenced by patronage patterns, institutional resilience, and the symbolic significance of spatial positioning. For instance, GIS-based analyses of temple locations in Xiamen have illustrated how both natural and constructed environmental factors influenced monastic expansion from the Sui–Tang through the Ming–Qing periods, revealing a consistent correlation between geographic placement and institutional prominence (Fu et al., 2025). Similarly, Ju’s (2023) study on the temporal and spatial distribution of Buddhist architecture in Zhejiang demonstrates that transportation networks, urbanization, and the diffusion of religious culture played significant roles in temple placement. Broader trends in Chinese scholarship, which emphasize the impact of political climates, socioeconomic development, natural geography, and heritage policies on temple siting, further reinforce these findings (Gao et al., 2024). Even in non-Han regions such as Amdo, proximity to rivers and elevation patterns have been shown to structure monastic landscapes in comparable ways (Fang et al., 2022), suggesting a geographically coherent logic across diverse Buddhist contexts.

Despite recent advancements, current research reveals several critical gaps. A significant portion of the literature focuses on the empirical mapping of temple distributions but fails to engage with the symbolic, performative, and political dimensions of sacred geography. Sacred space is often treated as a static site for ritual rather than a dynamic nexus where religious practices intersect with political power, cultural memory, and evolving territorial imaginaries. For example, studies of major sacred mountains like Mount Wutai and Mount Putuo predominantly emphasize mythological or cosmographic frameworks, neglecting the examination of their geopolitical and administrative roles (Tang and Wang, 2023). This oversight has profound implications for the study of Lingyan Temple. Despite its strategically significant location at the confluence of cultural and political corridors in the Jinan region, existing scholarship fails to critically explore how this spatial positioning influenced the temple’s long-term institutional authority, reputation, and ritual centrality. Such omissions reflect a broader trend that neglects the interplay between sacred geography and the complex socio-political forces that shape religious institutions.

Addressing these gaps necessitates a methodological shift. A historiographical and spatial-historical approach is essential not only to contextualize sacred sites such as Lingyan Temple within their broader geographic and political contexts but also to reconstruct their dynamic spatial trajectories over time. By combining spatial analysis with institutional history, this study makes a significant contribution to the scholarly discourse: it reconceptualizes Lingyan Temple not merely as a static ritual site but as a dynamic spatial entity, whose geographic location both influenced and was influenced by political transformations, cultural flows, and monastic networks. This integrated approach enables the present study to advance contemporary Chinese scholarship by shedding light on dimensions of spatial agency that have been inadequately theorized in existing research.

### Chronology and the cyclical development of temples

Buddhist temple institutions across East Asia—from China to Japan and Korea—typically undergo recurrent cycles of establishment, prosperity, decline, and renewal. Earlier scholarship has identified these institutional rhythms and loosely correlated them with dynastic transitions, fluctuations in political patronage, or waves of suppression (Dagiev, 2013; Zürcher, 2014). Yet, much of this literature presents only fragmentary or generalized chronologies, lacking the methodological precision required to reconstruct temple histories with verifiable accuracy. Narrative accounts frequently foreground miraculous origins or charismatic figures while omitting empirically demonstrable events such as construction phases, destruction episodes, and successive rebuilding (Landry, 2023). Foundational work in historical geography and the broader sinological study of temple institutions has long emphasized the need to correlate textual references with spatial and material evidence, but such principles are often applied inconsistently. Archaeological excavations, epigraphic materials, and authenticated documents offer crucial datasets: inscriptions provide explicit construction and restoration dates, whereas archaeological strata supply a stratified sequence of material development (Karkanas and Goldberg, 2018; Watson, 2022). Despite these resources, few studies integrate them into a coherent historiographical methodology grounded in systematic verification. Instead, chronologies typically remain siloed, creating significant gaps in interpreting institutional life cycles. The case of Lingyan Temple epitomizes this challenge: reconstructing its development—from its Eastern Jin foundation, flourishing in the Northern Dynasties and Tang, decline in later periods, and its eventual modern revival—requires a rigorously positivist synthesis of archaeological, epigraphic, and textual corpora, ensuring that each historical inference is anchored in demonstrable evidence.

Chinese Buddhist historiography traditionally centers on bibliographic materials, including dynastic histories (shi), temple gazetteers (sizhi), stele inscriptions, and monastic biographies (gaoseng zhuan). These sources provide indispensable information on institutional foundations, eminent monastics, ritual lineages, and networks of patronage (Zacchetti and Palumbo, 2022; Yang and Yang, 2025). However, they pose well-known methodological complications: gazetteers often served political, municipal, or economic ambitions; inscriptions regularly adopted hagiographic rhetorical styles; and dynastic histories, though official, frequently filtered events through imperial ideology. Scholars have long acknowledged these limitations, yet most employ such materials descriptively rather than subjecting them to systematic source criticism. In contrast, recent methodological advances in epigraphic analysis—such as stratigraphic cross-checking, palaeographic dating, and verification against archaeological horizons—have demonstrated that inscriptions can be used as rigorously as any archival document when treated through established best practices. Likewise, comparative sinological research on Buddhist temples in Japan and Korea shows that integrating inscriptions, archaeology, and textual records yields more reliable institutional chronologies than approaches relying on narrative sources alone.

Responding to these methodological debates, this study adopts an empirical, positivist historiographical framework while also engaging critically with contemporary discourse on historical positivism itself. Critics of positivism argue that strict verification can obscure interpretative nuance, flattening the cultural and symbolic layers of religious institutions. Yet proponents counter that objectivity, when carefully theorized, enables historians to distinguish demonstrable events from hagiographic embellishment, thereby clarifying the very conditions under which religious narratives emerge. By situating this study within these debates, the methodological choices employed here—triangulating bibliographic sources, epigraphy, and archaeological data—are justified not as naïve empiricism but as a historically grounded practice of disciplined verification. This approach allows for a systematic separation of narrative and evidence, ensuring that historiography does not simply reproduce inherited biases. Applied to Lingyan Temple, this framework treats its abundant bibliographic traditions as sources requiring layered evaluation: temple gazetteers and dynastic histories are examined for ideological framing and omission, while inscriptions are analyzed alongside archaeological findings to establish reliably dated events. Ultimately, this study demonstrates how Buddhist temple historiography can transcend descriptive reproduction by integrating critical source analysis with verifiable evidence, producing a more coherent and methodologically defensible reconstruction of Lingyan Temple’s institutional history.

## Methods

### Research approach

This study employs a positivist historiographical framework designed to reconstruct the institutional development of Lingyan Temple through systematic verification of material, textual, spatial, and oral data. Positivist historiography, following Gill et al. (2018) and Kala (2025), requires that historical claims be corroborated through multisource triangulation, critical analysis of source production, and replicable analytical procedures. This approach addresses long-standing issues in Buddhist historiography, namely the uncritical dependence on gazetteers and monastic biographies, by grounding the reconstruction in epigraphic and archaeological evidence as primary data, while textual and oral materials serve as secondary layers requiring evidentiary validation.

### Data collection

The study integrates three major datasets. First, the epigraphic corpus comprises 420 stone inscriptions from the Tang through the Republic era. All inscriptions were digitally photographed, transcribed, palaeographically dated, and coded according to temporal, architectural, and patronage variables. Second, textual data include the Li (1696), dynastic histories (*Jiu Tang Shu*, *Wei Shu*), monastic biographies (*Gaoseng Zhuan*, *Xu Gaoseng Zhuan*), and local gazetteers. Each document underwent historiographical criticism to evaluate internal consistency, political or sectarian bias, and embedded editorial agendas. Third, oral data were collected through semi-structured interviews conducted from March 2023 to January 2024 with 18 participants (monastic leaders, temple administrators, heritage officials, archaeologists, and local historians), see in Table 1. Participants were recruited through purposive sampling with maximal variation to ensure representation of ritual, administrative, conservation, and scholarly perspectives. Interviews lasted between 45–90 min, were audio-recorded with consent, and transcribed verbatim.

**Table 1:** Interview participants and characteristics.

Participant code	Role / position	Institutional affiliation	Years of experience	Interview date	Duration	Key contribution to study
TA-01	Abbot / Monastic Leader	Lingyan Temple	22 years	14 Apr 2023	78 min	Ritual continuity, institutional memory post-1949
TA-02	Senior Monk	Lingyan Temple	15 years	28 Apr 2023	63 min	Monastic practices, structural changes in temple routines
TA-03	Junior Monk	Lingyan Temple	6 years	12 May 2023	52 min	Daily rituals, contemporary challenges in monastic recruitment
ADM-01	Administrative Manager	Lingyan Temple	12 years	4 Jun 2023	71 min	Administrative archives, donation records, temple governance
ADM-02	Financial Officer	Lingyan Temple	8 years	18 Jun 2023	55 min	Patronage patterns, government–temple financial relations
HA-01	Senior Heritage Officer	Shandong Provincial Heritage Bureau	18 years	2 Jul 2023	84 min	State policies on conservation, restoration procedures
HA-02	Heritage Conservation Architect	Provincial Restoration Office	11 years	20 Jul 2023	69 min	Architectural stratigraphy, reconstruction documentation
LH-01	Local Historian	Jinan Historical Society	27 years	6 Aug 2023	82 min	Local archival history, oral transmission of temple legends
LH-02	Cultural Historian	Mount Tai Cultural Research Institute	19 years	22 Aug 2023	76 min	Regional religious system, historical spatial networks
LH-03	Independent Researcher	Taishan Cultural Association	14 years	5 Sep 2023	58 min	Epigraphic interpretations, lineage narratives
GOV-01	Municipal Cultural Official	Jinan Culture and Tourism Bureau	9 years	18 Sep 2023	64 min	Tourism development, heritage–economy tensions
GOV-02	Regional Planner	Tai’an Regional Planning Office	7 years	30 Sep 2023	49 min	Spatial planning, landscape–heritage integration
EF-01	Elder Lay Practitioner	Lingyan Temple Lay Community	33 years	11 Oct 2023	57 min	Ritual memory from pre-reform to reform era
EF-02	Lay Archivist	Taishan Buddhist Association	21 years	25 Oct 2023	65 min	Manuscript preservation, gazetteer circulation
EF-03	Stone Inscriptions Collector	Local Epigraphic Society	12 years	9 Nov 2023	54 min	Epigraphic condition, missing fragments documentation
AR-01	Archaeologist	Shandong University	17 years	26 Nov 2023	80 min	Stratigraphic data, archaeological phases of the temple
AR-02	Field Archaeologist	Institute of Cultural Relics	10 years	14 Dec 2023	72 min	Validation of construction layers, artifact dating
AR-03	Palaeography Specialist	Chinese Academy of Social Sciences	16 years	8 Jan 2024	73 min	Calligraphic dating, inscription chronologies

#### Thematic analysis

Interview data were analyzed using thematic analysis based on Braun and Clarke (2006, 2019). The process included familiarization with transcripts, inductive coding in NVivo 14, development of preliminary themes, iterative theme review, definition and refinement of themes, and final synthesis. NVivo 14 facilitated node comparison, cross-linking of interview themes to epigraphic data, and consistency checks between oral accounts and documentary sources. Themes such as ritual continuity, restoration memory, patronage transitions, and institutional longevity were retained only when substantiated by at least one material or textual dataset, ensuring alignment with the positivist requirement of empirical corroboration.

#### Triangulation and verification

All themes were systematically cross-validated with inscriptional chronologies, archaeological stratigraphy, and textual evidence. Conflicting narratives were resolved through palaeographic dating, architectural layering analysis, and reign-year calibration. Translation of Classical Chinese inscriptions was performed independently by two epigraphists, with inter-rater reliability *κ* = 0.82; discrepancies were resolved through joint review and back-translation. Spatial-historical analysis used historical cartography, topographic interpretation, and geospatial comparison to situate institutional development within regional networks. This multi-stage verification process ensured that no historical claim relied solely on oral or textual testimony without support from material evidence.

## Results

*Findings from the first research question (RQ1): how do geographical factors influence the historical development and strategic position of Lingyan Temple from the eastern Jin period to the modern period?*

The geographical influence on the historical development of Lingyan Temple cannot be separated from the classical Chinese cosmological framework and the regional administrative structure that existed from the Eastern Jin to the Tang Dynasty. Its location on the southwestern slope of Mount Tai (Tai Shan) was not simply a pragmatic geographical choice, but a meaningful religious and political decision. Mount Tai is one of the Five Sacred Mountains of China and was a key location for imperial inauguration ceremonies (fengshan), giving the surrounding area a spiritual status. Since Lingyan Temple is located on the northwestern side of Mount Tai, it is important to highlight this location in particular in order to understand when Buddhism was introduced to the Mount Tai area and how the local people received it. Historical evidence suggests that Buddhism entered the Shandong region as early as the first century AD. Figures such as Liu Ying are recorded as the first Buddhists in Shandong, while Ze Rong’s religious practice flourished in the second century. Particularly intense Buddhist activity was found in Pengcheng, an important centre at that time due to its location at the eastern end of the Silk Road, the main route through which Buddhism spread to China. In contrast, the Mount Tai area was not on the main trade route and was far from Luoyang, the capital of the Eastern Han Dynasty, which was also the centre of Buddhism at that time. Therefore, there is no direct record of the spread of Buddhism in Mount Tai before the third century.

The earliest record linking Mount Tai to Buddhism is found in Kang Senghui’s translation of the Liudu jijing (六度集經) in the third century. In this work, he equated the concept of “hell” in Buddhism with Mount Tai, since the Chinese at that time believed that Mount Tai was the final resting place of the dead. This is supported by inscriptions found on tombs from the Eastern Han Dynasty that read, “the living go to Chang’an in the west, the dead go to Mount Tai in the east.” In the ancient Chinese view, Mount Tai was a great and majestic mountain, often called “太山” (Tai Shan) to emphasise its grandeur. A famous scholar from the Qing Dynasty, Gu Yanwu, noted that the belief in Mount Tai as a place for spirits and ghosts originated in the late Han Dynasty.

In contrast, the belief in the existence of immortal gods had appeared since the late Zhou Dynasty. Statistics in the Liudu jijing show that “Mount Tai” appears 28 times, “Mount Tai as hell” appears 9 times, and the term “hell” itself appears 3 times. In later translations of Buddhist texts (from the Three Kingdoms to the Five Dynasties), using “Mount Tai” as a metaphor for hell became a common practice. Although it is unknown to what extent the local people of Mount Tai accepted the assimilation of Buddhism and local folk beliefs, this can be considered the starting point of Buddhism’s penetration into the region.

*The Geographical and Topographical Influence of Mount Tai on the Structure and Development of Lingyan Temple*

In order to understand the geographical factors that influenced and shaped the Lingyan Temple, it is first necessary to define what “region “means. There are various opinions regarding how to define the boundaries of a region. Zhou Zhenhe 周振鶴 once stated that “marking a region using administrative units, especially contemporary provincial boundaries, has obvious advantages” because “administrative boundaries are formed as a result of negotiations between various forces in history and are based on the geographical characteristics of the region, taking into account many political, military and economic aspects.” However, Dr. Jiang Wu disagrees with Zhou’s view. He emphasizes that administrative units are “not suitable for the study of religious sites” and that “physiographic regions formed by physical geography and connected by traditional transportation routes provide an alternative.” Dr. Wu used Skinner’s nine macro-regions of China in his research. However, since Dr. Wu’s research covers the whole of China while my research only focuses on one Buddhist temple in China, Skinner’s physiographic macro-regions seem too broad. Therefore, I chose to use “Shandong” as the study area in this research, which was initially a geographical concept and later became an administrative division in Chinese history. “Shandong” as a geographical concept has long been known in Chinese history.

The emergence of the term “Shandong” can probably be traced back to the Warring States (Zhanguo) period. Guan Zhong, a famous philosopher and statesman of that period, once said in Guanzi that “the State of Chu is a powerful state in Shandong” (Guanzi, as quoted in Wu, 2022). However, Guan Zhong’s understanding of “Shandong” differs from that of the present-day administrative unit of Shandong. What he meant was a vast area east of the Taihang Mountains. The meaning of “Shandong” changed when China became one country during the Qin and Han dynasties. In a general sense, the word still means the area east of the Taihang Mountains. In a more specific sense, it refers to the area that is currently part of Shandong Province’s administrative boundaries. Sima Qian, a great historian of the Han Dynasty, used the term “Shandong” in a narrow sense in several places in the Shiji. For example, in the Rulin Liezhuan chapter, the story of Fu Sheng, a Confucian scholar who hid the Shang Shu manuscript during the book burning by Qin Shi Huang, is told. Fu Sheng got the manuscript back when the Han Dynasty came back to power, but some of it was missing. He then taught it to his students in the Qi and Lu regions [Sima, as cited in Watson (2022)]. Watson’s translation of “Shandong” as “east of the mountains” indicates that he adhered to the conventional geographical interpretation. However, recent studies have shown that the term in the text refers to the narrow sense, referring to the Qi and Lu regions, the regions now synonymous with Shandong (Wu, 2022). Thus, despite the differences in interpretation, the term “Shandong” has been a widely accepted geographical concept since the Han Dynasty. As an administrative division, “Shandong” was officially designated in 1168 during the Jin Dynasty, and continued to be used in the Yuan, Ming, and Qing Dynasties.

Along with consolidating the Shandong region as an administrative and cultural entity, the region also experienced significant symbolic and spiritual development. Traditions and cultural heritages that had been rooted since prehistoric times provided the foundation for developing a religious landscape, which later became important in Chinese Buddhism. In this process, geographical and cosmological elements were physical, forming a sacred basis for spreading religious teachings and practices. Buddhism spread to the Shandong region not long after it spread to China. According to a study by Erik Zürcher, “by about the middle of the first century AD, Buddhism seems to have spread to the region north of the Huai River, including eastern Henan, southern Shandong, and northern Jiangsu.” Zürcher’s conclusion is based on the record in the Hou Han Shu 後漢書, which states that the Ming Emperor issued an edict in 65 CE to Liu Ying 劉英, King of Chu 楚, which read: “the king of Chu reads the gentle words of Huanglao 黃老’s teachings, and respectfully makes gentle offerings to the Buddha.” Liu Ying’s kingdom of Chu was located in Pengcheng 彭城, which covered most of Jiangsu and Shandong during the Eastern Han Dynasty.

These records show that Buddhist activity had occurred in Shandong since the first century CE, a finding that deserves further investigation. Why did Buddhist activity occur in Pengcheng and not elsewhere? The main reason lies in Pengcheng’s strategic location. As Zürcher explains, “Pengcheng was a thriving trade centre located on the main route from Luoyang to the southeast, an eastern extension of the continental Silk Road through which foreign traders from the West used to travel. Furthermore, to the northwest, Pengcheng was connected with Langya 瑯琊 in southern Shandong, and to the southeast with Wujun 吳郡 and Kuaiji 會稽, important maritime trade centres that were connected via Panyu 潘禺 (Guangzhou) to ports in Indo-China and the Malay Peninsula.” Pengcheng’s unique geographical location certainly provided favourable conditions for the spread of Buddhism, as it served as a hub for trade and cross-cultural mobility. It is unsurprising to find Buddhist activities, even Buddhist sites, in the area. The Shuijing zhu 水經注 records that an Ashoka Temple (阿育王寺) was established in Pengcheng during the reign of Liu Ying. Although some scholars doubt the possibility of a temple named after King Ashoka at such an early period, the existence of records in the Shuijing zhu at least suggests that there was one or more Buddhist sites used for religious activities, given that Liu Ying was known to be a devout Buddhist.

The early development of Buddhist activity in southern Shandong and its connection to major trade routes provide a strong historical background for understanding how Buddhism spread to Mount Tai. With its sacred and symbolic location, the area became an important node in the spread of Buddhism in China. The Mount Tai (Tai Shan) area has had a strong spiritual and symbolic significance in Chinese culture, even before the advent of Buddhism. As one of the Five Sacred Mountains (五岳, Wuyue), Mount Tai is associated with the fengshan (封禅) ritual, a religious and political ceremony performed by emperors to strengthen their legitimacy through symbolic communication with heaven and earth. Lingyan Temple is located on the southwest slope of a mountain, making it not only a local religious site but also an important part of China’s religious power and cosmological landscape. The location of Lingyan Temple has advantages in terms of its topography, structure, and spirituality. The temple is built to fit the natural shape of the mountain, creating a vertical space that represents the spiritual journey from the lower world to enlightenment at the top. In Mahayana Buddhist architecture, this design is not only decorative; it is also a physical representation of the cosmological beliefs of samsara and nirvana. The ascending pilgrimage path, from the main gate to the main hall at the top of the complex, is designed to create a gradual transcendental experience for pilgrims and monks.

Its strategic location on the route between Jinan (the regional capital) and the religious centre of Mount Tai made Lingyan Temple a spiritual transit point and a centre for Buddhist learning. From the Jin to the Tang dynasties, ancient maps and geospatial analyses show that the temple was at the center of regional communication and transportation networks. The discovery of stone inscriptions from the Tang and Song dynasties that record visits and donations from military and civil officials for temple renovation backs this up. Sacred spaces like meditation pavilions and sutra reading rooms were also affected by ecological factors like mountain streams, old trees, and morning sunlight. The layout follows feng shui rules, which try to balance spiritual energy with the natural world. The temple’s architecture also adapts to the terrain: terraced buildings follow the slope, and stone paths are arranged to harmonise with the surrounding natural elements.

From a functional perspective, the hilly geographical conditions also provide natural defensive advantages. This explains why Lingyan Temple is relatively more resistant to military attacks or disasters than temples in the plains. Many temples were destroyed during political turmoil, such as the late Tang and early Song dynasties, but Lingyan remained an important religious centre in Shandong. Thus, selecting the location of Lingyan Temple was not a random decision, but rather the result of a synthesis of cosmological values, ecological conditions, and religious administrative strategies. A geographical perspective on the historiography of Lingyan Temple indicates that its existence transcended mere religious function, evolving into an institution shaped by the interplay of the natural environment, political authority, and regional religious networks.

In Chinese Buddhism, the temple’s position in this area creates a symbolic connection between the human world (earth) and the transcendent world (heaven or heaven), per the doctrine of mandala and axis mundi in Mahayana Buddhist cosmology. The placement of Lingyan Temple in this landscape provides spiritual value and reflects political and administrative strategy. Through spatial-historical analysis of satellite imagery and historical topographic maps, it was determined that Lingyan Temple is located along the ancient route connecting the historical power center of Luoyang with the provincial capital of Jinan. This route, part of the military and religious transportation network during the Tang period, facilitated the mobility of monks, imperial officials, and elite donors who were the principal patrons of the temple’s construction.

For instance, an inscription from 858 AD talks about how a military governor of Jinan used his own money to rebuild the Pizhi Pagoda after having a religious dream vision. This implies that the temple’s location facilitated a direct link between spiritual influence and administrative backing. Zeng (2022) assertion underscores the significance of the Regional Religious System in elucidating the persistence of religious institutions like the Lingyan Temple beyond the direct oversight of the central state. The Lingyan Temple’s geospatial aspect can be observed not only through administrative connections but also through the internal configuration of the temple complex. Based on historical plans from the Song and Ming dynasties compared with modern archaeological documentation, it can be seen that the layout of the main hall, pavilions, and pagodas follows the natural contours of the mountain. This saves resources and creates a dynamic experience of sacred space.

The vertical approach to the temple’s construction, from the entrance in the valley to the spiritual summit of the main hall, reflects the soteriological journey to enlightenment. Visually, this gives the effect of pilgrimage elevation, a gradual transcendental experience through space and elevation. Other environmental factors, such as mountain water flow, natural vegetation, and sun-facing positions, were considered in the temple’s structural construction. This principle is consistent with the Chinese practice of geomancy (fengshui), which aims to balance spiritual energy in physical space. Thus, the integration of temple architecture and geographical landscape is not simply a technical decision, but an expression of the theology of space in Chinese Buddhism.

*Findings from the second research question (RQ2): How can the chronological structure of the establishment, progress, decline, and revitalisation of the Lingyan Temple be reconstructed based on verified archaeological evidence, inscriptions, and historical documents?*

*Early Establishment and Growth (Wei, Jin, Southern and Northern Dynasties)*

The Six Dynasties (Wei, Jin, Southern and Northern Dynasties) are considered a critical period in the early development of Buddhism in China. Buddhism spread widely to various regions during this period and profoundly influenced Chinese philosophical thinking. One of the main factors that accelerated this spread was the translation of Buddhist scriptures. Since the Han Dynasty, several foreign monks have come to China and contributed significantly to translating Buddhist texts. Among them are names such as An Shigao and An Xuan from Anxi (now Iran), Lokaksema and Zhi Yao from the Kushan region, Zhu Foshuo from Tianzhu (India), and Kang Mengxiang from Kangju.

During the Three Kingdoms period, monks such as Tan Kejia Luo, Tandi, and Kang Sengkai actively translated scriptures in the Cao Wei region, while in the Dong Wu region, this activity was carried out by Wei Zhinan, Zhi Qian, and Kang Senghui. During the Western Jin Dynasty, the most influential translator before Kumarajiva, Dharmarakṣa (Zhu Fahu), produced more accurate and understandable translations, thereby expanding the reach of Buddhism in Chinese society. In parallel, the turbulent socio-political conditions of the period strengthened the appeal of Buddhism. After the collapse of the Eastern Han Dynasty in 220 CE, China experienced a period of chaos for more than three centuries before being reunified by the Sui Dynasty in 581. The fragmented political situation and prolonged civil war left the people uncertain and suffering. In this context, Buddhism became a source of spiritual comfort. Its teachings on karma and salvation after death gave the suffering ordinary people new hope.

Meanwhile, scholars were attracted to Buddhism because they found similarities between Buddhist teachings and the philosophy of “xuanxue” (玄學, or Dark Learning) popular among Jin intellectuals. Both schools discussed the concepts of emptiness and non-existence. As Zürcher (1959) points out, the popularity of discussions on “emptiness” and “nothingness” became an important factor in the development of Buddhism among the Chinese nobility and scholars in the early period. In addition to being accepted culturally and philosophically, Buddhism also gained political legitimacy from the rulers. Emperors and officials began to support projects that translated scriptures and built temples. This support made Buddhist scriptures an important means of penetrating Buddhism into the socio-political structure of China. At the local level, the development of Buddhism was also prominent, although at an uneven level in each region.

During the Han and Three Kingdoms dynasties, monasteries were built primarily to provide places of worship and accommodation for foreign monks and merchants. However, from the Jin Dynasty on, monasteries began to be supported by the court and local nobility. This transformation brought about a significant change in monastic life. Monks were no longer nomadic but settled in monasteries, practising their religion and spreading Buddhism from within a permanent religious institution. Historical data also shows the disparity in the construction of monasteries between regions. According to records in the Bianzheng Lun (辯正論), there were about 180 monasteries in Luoyang and Chang’an during the Western Jin Dynasty, with the number of monks and nuns reaching more than 3,700. The Luoyang Jialan Ji recorded the existence of 42 monasteries in Luoyang during the reign of Emperor Yongjia. Meanwhile, based on a study of local gazetteers, 58 new monasteries were established during the Western Jin period and spread across 12 of the 19 zhou (administrative provinces).

This map of the distribution of temple construction shows that areas such as Yang Zhou had more than half of the new temples (31), while the Shandong region, covering Ji Zhou and Yan Zhou, recorded only one or two temples each. This indicates that the spread of Buddhism in Shandong during the Jin period was not as advanced as in other regions. However, it is believed that Lingyan Temple was established as part of the local expansion of Buddhism during the Eastern Jin period. Primary documents such as the Gaoseng Zhuan (高僧傳), Biqiuni Zhuan (比丘尼傳), and Wei Shu (魏書) record Buddhist activities in areas such as Langya (modern Linyi), Mount Tai, and Rencheng. This supports the argument that although Shandong was not the initial centre of Buddhism, it later played a significant role in the phase of the spread and institutionalisation of Buddhism, of which Lingyan Temple was one of the most important manifestations.

Geographically, more than half of the Buddhist temples built during the Western Jin Dynasty were located in Yangzhou, indicating that this region rapidly developed as a centre for the spread of Buddhism, even competing with Luoyang and Chang’an as major religious centres. Jingzhou also stands out, with eight new temples, most believed to have been located in Xiangyang. In contrast, the number of new temples recorded in Luoyang (Sizhou) and Chang’an (Yongzhou) is relatively small. This may be because many temples were built before the Western Jin period and were not included in the new construction data. If we look at the Shandong region, we find that the development of Buddhism there was much slower than in other regions.

Administratively, Shandong covers Qing Zhou and most of Yan Zhou, as well as parts of Ji Zhou (northwest), Yu Zhou (southwest), and Xu Zhou (south). According to the data, no new temples were built in Qing Zhou, Yu Zhou, and Xu Zhou during the Western Jin, with only two temples in Ji Zhou and one in Yan Zhou. This disparity suggests that regional factors were significant in forming Buddhist centres. It was not until the Eastern Jin that Shandong experienced significant Buddhist growth, including the establishment of the Lingyan Temple. The distribution of Buddhist activities in Shandong during the Jin Dynasty shows a significant change: while in the Western Jin, only Langya was religiously active, during the Eastern Jin and Sixteen Kingdoms periods, Buddhist activities spread widely. Primary texts such as the Gaoseng Zhuan (GSZ), Biqiuni Zhuan (BQNZ), and Wei Shu (WS) record a sharp increase in the number of monks and nuns, especially in the Mount Tai area. This region is widely recognised as Shandong’s most important Buddhist centre. One of the leading figures who contributed to this was the monk Zhu Senglang (竺僧朗), the founder of Lingyan Temple.

Zhu Senglang’s biography in the Gaoseng Zhuan describes him as a holy figure whom the people and rulers across dynasties revered. After searching for the proper way (dao), he settled on Mount Tai and built a jingshe (spiritual hut) in Jinyu Valley, Mount Kunlun, northwest of Mount Tai. He attracted hundreds of followers and gained the support of emperors such as Fu Jian of the Former Qin, Yao Xing of the Later Qin, Murong De of Yan, and Emperor Xiaowu of the Eastern Jin and Tuoba Gui of the Northern Wei. Emperor Fu Jian even issued a special edict to exempt Senglang from the examination of fake monks because of the purity of his practice.

Zhu Senglang was known to have extraordinary spiritual abilities, including accurate predictions and a spiritual influence that could calm the surrounding wild animals. The area where he lived was later named Langgong Valley. Before his death at the age of 85, he succeeded in making the area a respected centre of Mahayana practice, marking the birth of Lingyan Temple as an important Buddhist institution in the Shandong region. Zhu Senglang’s religious, social, and symbolic contributions not only strengthened Mount Tai’s position as a sacred centre but also bridged the transition of Buddhism from a nomadic practice to a settled temple system typical of China.

### The reestablishment of Lingyan Temple

Although Lingyan Temple was not supposed to be directly affected by the first Buddhist persecution in 446 CE, historical records show that it underwent a phase of reestablishment during the Northern Wei and Southern Wei dynasties. This contradicts previous assumptions about Lingyan Temple’s non-involvement in the first wave of state destruction of Buddhist temples. The temple’s only extant Gazetteer, the Lingyan Zhi (LYZ), records the historical development of Lingyan Temple as follows:

"Lingyan Temple is located at the foot of Mount Fang, which, in the Shui Jing, is referred to as 'Mount Yufu is one of the hills in the northwest range of Mount Tai'. During the reign of Fu Jian, the name of the valley where the temple is located was changed to Jinyu Valley of Mount Kunlun in honour of Langgong, a prominent Buddhist figure who spread Buddhism in the area. This location is under the administrative jurisdiction of Shanchi County, Taishan Prefecture. In the seventh year of the Taiping Zhenjun era (446 AD), Emperor Taiwu of the Northern Wei Dynasty issued an edict to destroy Buddhism, ordering the slaughter of all monks and the destruction of temples, and the reversion of Jinyu Valley to Mount Fang, its previous name.

The original text is 靈岩寺在方山下, 即《水經》:“玉符山, 乃泰山西北麓之一岩也。“符秦時改名 昆侖山金輿谷, 蓋重其人而神其地也。時朗剬在此說法。屬泰山郡山茌縣。元魏太武帝太平真君七年, 詔誅天下沙門, 毀佛寺, 革昆侖金輿之名, 仍名曰方山, 至今因之。元魏孝明帝正光初, 法定禪師先建 寺于方山之陰, 曰“神 寶”。後建寺于方山之陽, 曰“靈岩” (See Ma, 1998, Lingyan Zhi 灵岩志, 22)

This quote shows that the location of Lingyan Temple underwent several name changes, namely Fang Mountain, Yufu Mountain, and Kunlun Mountain. In the Lingyan Zhi, the first temple built by Zhu Senglang in the Jinyu Valley is identified as Lingyan Temple. However, various other historical sources question this claim, stating that the first temple built by Langgong was Shentong Temple or Langgong Temple. Although Langgong most likely founded Lingyan, it was not in the exact location and was built at a different time from Shentong Temple. The Lingyan Zhi records that Lingyan Temple was destroyed in 446 AD during the great persecution of Buddhism by Emperor Taiwu. However, there is a flaw in this historical logic, because the Qi Zhou region, where the temple was located, was still controlled by the Liu Song Dynasty until 467. This means that the Northern Wei Dynasty’s edict to destroy temples should not have been in effect in the region in the year in question.

The lack of written records about Lingyan Temple during the Northern and Southern Dynasties makes the remaining stone inscriptions an important source of information. One of the most valuable inscriptions is the “Lingyan Si Song Bei” (Inscription of Praise of Lingyan Temple) from the Tang Dynasty, which records the early history of Lingyan Temple. According to the Jinshi Lu by Zhao Mingcheng of the Song Dynasty, this inscription was written in xingshu (running script) style by Li Yong on November 15, 742 AD, while he was the governor of Beihai in Shandong. Unfortunately, the inscription was later damaged and illegible. When the Lingyan Zhi was compiled in 1696, it was found half-buried in the ruins of Shenbao Temple. Later, when the Shanzuo Jinshi Zhi was compiled by Ruan Yuan in 1793, the inscription had been broken into two and displayed on the wall of the Luban Cave in the temple complex. It was eventually reprinted in the Baqiongshi Jinshi Buzheng by Lu Zengxiang, and a scraped copy of it was published in the 1999 book Lingyan Monastery.

The records show Master Fading’s activities [noted in *Gaoseng Zhuan* 高僧傳 (Huijiao, 1987) and *Xu Gaoseng Zhuan* 續高僧傳 (Daoxuan, 645/1988)] around the early 5th century, after the Eastern Jin’s fall and the Liu Song’s rise in 420 CE. However, the LYZ states that Lingyan Temple was rebuilt in 520. This means that if Master Fading were active in 420, he would have been over a hundred years old by the time of the rebuilt event—a highly unlikely, though not impossible, possibility in Buddhist hagiography. Therefore, scholars such as Wen Yuxian have concluded that Shenbao Temple was built in 520, not Lingyan Temple. However, the LYZ again states that Shenbao was built before Lingyan, further confusing the chronology. Religious legends further compound this confusion by reinforcing the temple’s sacred status through supernatural narratives. For example, in the story of Master Fading, two silk-bearing tigers and a green snake are said to have guided him to the site of the temple’s founding. Sunlight opened a cave on the mountaintop, water gushed out after being poked with his stick, and creatures such as white cranes and yellow monkeys appeared as if giving divine signs that the place was worthy of being a holy site. This legend is similar to the narrative of Langgong’s founding of Lingyan Temple, where the stones are said to have nodded as he recited sutras.

These narratives are consistent with Bingenheimer’s argument that the founding legend of a sacred site functions as an origin myth, binds the site to the religious imaginary, and forms local legitimacy. In the case of Lingyan, associations with sacred figures such as Langgong and Fading, coupled with miracle stories, played a significant role in shaping the “holiness” of the site—especially in a highly competitive religious landscape such as the Mount Tai region. Thus, although textual evidence strongly suggests that Master Fading was a key figure in the reconstruction of Lingyan Temple around 420, contradictions between the LYZ and other sources and the strong influence of religious legends make the history of the temple’s reestablishment difficult to establish empirically. Nevertheless, the integration of epigraphic data, historical narratives, and oral accounts suggests that the reestablishment of Lingyan Temple was not a single event, but rather a multi-layered process that took place in the complex socio-political and spiritual contexts of the Southern and Northern Dynasties.

#### Modern era: post-reform and contemporary developments

After suffering extensive damage during the Cultural Revolution (1966–1976), Lingyan Temple entered a significant rehabilitation phase during China’s reform era under Deng Xiaoping. Along with the policy of openness and religious restoration, the government began to allow the reopening of previously closed religious sites. Lingyan, which had lost many of its artefacts and physical structures, became one of the restoration priorities in Shandong Province. In 1983, the People’s Republic of China government officially recognised Lingyan Temple as one of the ‘National Buddhist Sacred Sites’, paving the way for structural and institutional restoration.

According to one temple administrator,

*“During the 1980s reconstruction, our main concern was not only rebuilding structures but restoring the spirit of the site. Many of the old monks emphasized that the temple must ‘breathe with the mountain again’ after the disruptions of the Cultural Revolution”* (ADM-01 Interview).

The Cultural Revolution significantly altered the layout of Lingyan Temple, resulting in the destruction of statues and rooms. The demolition of smaller shrines and meditation pavilions disrupted the geomantic alignment of the complex, which had traditionally been oriented along the south–north spiritual axis. When restoration efforts commenced in 1983, they adhered to state-prescribed guidelines for architectural preservation, rather than the original fengshui principles. As a result, while the new layout restored the temple’s visual integrity, it partially undermined the natural harmony between the ritual axis, water flow, and mountain contours that had defined Lingyan’s spiritual space for over a millennium.

The religious revitalisation in Lingyan reflects the contemporary religious dynamics in China. According to an interview with Abbot Hong’en, with only seven permanent monks remaining, the main challenges are regenerating the monastic community and rebuilding the temple’s ritual functionality. However, with the growing interest of urban society in spiritual tourism, activities such as chanting, open meditation, and the Vesak festival are being revived. Traditional celebrations are combined with modern approaches.

A senior monk reflected that *“Even when the buildings suffered damage, the morning recitation never truly disappeared. A few elders maintained it quietly in their homes, and when rituals were revived in the 1990s, they guided the younger monks so the old rhythm would not be lost”* (TA-02 Interview).

The spatial boundary between sacred and secular zones has been further transformed in the modern era with the expansion of tourism infrastructure, including the construction of new access roads, a parking area, and commercial kiosks. Interviews with Abbot Hong’en and resident monks reveal that the contemplative atmosphere has been disrupted, and the “natural qi” that once flowed between the forested slopes and the pagoda ridge has been disturbed by the influx of visitors and commercial activities. Nevertheless, the monastic community has continued to reinterpret the site’s sacredness by promoting environmental awareness and reviving traditional rituals as a form of spiritual ecology. This evolving dynamic exemplifies the concept of a “sacred space in motion,” reflecting broader transformations within modern China (Table 2).

**Table 2:** Geographical findings of Lingyan Temple.

Geographical aspects	Description of findings	Historical implications
Topographical location	The temple is located on Mount Tai’s southern slope, considered a Holy Mountain in Chinese cosmology.	Strengthening the sacred status and increasing the long-term appeal of the temple
Regional accessibility	Close to the administrative centre of Jinan and located on the main route between Luoyang and Jinan.	Facilitating political and administrative patronage from regional rulers
Cosmological relationships	In the Mahayana Buddhist tradition, the location creates a symbolic connection between earth and sky.	Strengthening the religious identity of the temple as a cosmological axis
Integration with pilgrimage routes	It was a strategic stopping point for officials and monks on the pilgrimage route to Mount Tai.	Allowing the growth of temples as religious and cultural centres
Influence of natural environment	The natural topography supports vertical architecture and the spiritual experience of climbing the temple.	Connecting Buddhist doctrine with local values through spatial design and orientation

The rise of China’s economy has had a big effect on religious tourism. Lingyan Temple is now part of the “sacred circuit” of tourism on Mount Tai, drawing thousands of tourists from China and other countries every year. In Shandong tourism brochures, the local government calls Lingyan a “major spiritual and historical site” and tells stories that highlight its rich cultural and architectural value. But turning spirituality into a product also has its problems. Criticism has been raised about commercialising religious practices, such as the sale of amulets and high entrance fees. There is also debate about the temple’s autonomy in the face of administrative interference. Lingyan also plays a role in preserving ancient Buddhist manuscripts. Since 2010, a project to digitise the temple’s sutras and archives has been carried out in collaboration with local universities. Ancient documents such as the Lingyan Zhi and the list of monks’ biographies are used for academic research and public education. The project aims to preserve textual heritage and bring the public closer to the history of Chinese Buddhism. In the context of the Regional Religious System developed by Jiang Wu, Lingyan played an important role as a regional centre in the temple network in Shandong Province. Its geographical position at the foot of Mount Tai and its history of interaction with civil power made Lingyan an important node in the spatial dynamics of religion. Recent studies suggest that Lingyan was influential as a pilgrimage site and a ‘living archive’ of local religious and cultural history.

Table 3 shows the most important events in the rehabilitation and development of Lingyan Temple since the 1970s. It also shows how state policy, heritage conservation, and religious revival have all worked together since then.

**Table 3:** Chronology of rehabilitation and development of Lingyan Temple in the modern era.

Year	Important events	Information
1978	The beginning of the era of reformation and openness	The government begins to allow religious activities
1983	Recognized as a National Buddhist Holy Place	Official restoration begins
1990	Major architectural improvements (Hall and Pagoda)	Financial support from the provincial government
2005	Improvement of public facilities and religious tourism	Parking, information boards, pilgrim paths
2018	Waisak festival is celebrated nationwide	Broadcast on social media and local TV

This timeline shows that the temple’s recent growth still fits the idea of a “sacred space in motion,” where religious, political, and economic forces are always changing its institutional identity.

Lingyan Temple remained a regional monastic center through many dynasties, from the Tang reconstruction to the modern revitalization. During the Song and Jin periods, epigraphic evidence shows that both emperors and common people were still supporting the Pizhi Pagoda and copying Buddhist sutras, especially families of local gentry. During the Yuan and Ming dynasties, the temple’s purpose changed to include scholarly and memorial activities. This is shown in the Lingyan Zhi and in Ming-period monastic gazetteers that show how the temple was rebuilt many times after earthquakes. During the Qing dynasty, there was more of a focus on codifying texts than on building new ones. For example, the 1,696 edition of the Lingyan Zhi brought together earlier records into a single institutional history. The temple’s physical structure deteriorated after the late Qing, but its historical continuity was maintained through gazetteer transmission, safeguarding its sacred narrative into contemporary times.

#### A chronological reevaluation of Lingyan historiography

A positivist reevaluation of the aforementioned chronological discrepancies yields a more coherent reconstruction of Lingyan’s early development. First, the Lingyan Zhi’s record of the 446 CE destruction decree (Liu et al., 945/1975; Ma, 1998), cannot be historically supported because Liu Song was still in charge of the area at that time. The evidence indicates a subsequent period of damage occurring between 470 and 490 CE, coinciding with the Northern Wei’s eastern expansion. Second, the age difference of Master Fading shows that two historical figures or people who held the same clerical title in succession have been mixed up. This is backed up by the fact that stele inscriptions use different languages. Third, the assertion that Xuanzang translated sutras at Lingyan Temple in 629 CE is a Qing-era addition [*Daoxuan’s Xu Gaoseng Zhuan* 續高僧傳 (T50, no. 2060, 645 CE) and *Bianji’s Da Tang Xiyu Ji* 大唐西域記 Bianji (646/1985) (T51, no. 2087, ca. 646 CE)]; it was probably Xuanzang’s students who later contributed to the temple’s scholarly work around 650–660 CE. These changes fix the inconsistencies in the text while keeping the historical accuracy of Lingyan’s story, showing how useful it is to check facts from different sources.

*Findings from the third research question (RQ3): what are the characteristics of the primary bibliographic sources in compiling the historical narrative of the Lingyan Temple empirically?*

The study of the historiography of Lingyan Temple (Lingyan Si) requires the search for primary sources that can verify the temple’s historical narrative and institutional development. Various sources, such as stone inscriptions, gazetteers (zhi 志), monastic biographies, and administrative archives, provide an important foundation for understanding the temple’s transformation over time. One of the most important sources is the Lingyan Zhi (LYZ), compiled during the Kangxi Emperor’s reign (1696), the only complete Gazetteer still available today. An inscription from 858 CE, for instance, references the Jinan governor overseeing the reconstruction of the Pizhi Pagoda. This event is also documented in both the *Lingyan Zhi* (vol. 2, 1,696) and the *Tang Huiyao*, confirming its historical accuracy. The consistency across these sources indicates the use of positivist verification, whereby multiple lines of evidence corroborate the same historical event. All sources, including epigraphic, documentary, and legendary accounts, were evaluated according to rigorous empirical criteria to ensure consistency and reliability in interpretation.

Lingyan Temple has more than 420 stone inscriptions scattered around the temple grounds. These inscriptions were written by monks, officials, and scholars from various eras—from the Tang Dynasty to the Republic of China. In addition to inscriptions and gazetteers, other important sources are monastic biographical texts such as the Gaoseng Zhuan and Xu Gaoseng Zhuan, which record the lives of important monks such as Fading and Senglang. Researchers can construct a more objective historiographic narrative framework through triangulation between gazetteers, inscriptions, and manuscripts.

Local historical knowledge also contributes to understanding the temple’s fragmented inscriptions. As one historian explained, *“Families around the mountain preserved stories about the stele fragments long before formal documentation. Some elders even remembered where certain stones once stood before fires or landslides altered the terrain”* (LH-01 Interview).

The Lingyan Zhi Gazetteer was not only important as an official historical document, but also served as a means of institutional legitimacy for the temple in the eyes of the Qing authorities. Through Li Xingzu’s reworked narrative, many historical figures were symbolically associated with Lingyan’s greatness, although not all of these connections can be independently verified. This highlights that Buddhist historiography often operates on the boundary between fact and theological construction. On the other hand, epigraphic studies of the stone inscriptions at Lingyan provide access to firsthand data rarely touched by official revision. For example, inscriptions from the Song era specifically record the amount of donations, the origins of patrons, and even the political conditions of the time. This study finds that in some periods, such as the Southwest Jin invasion, temple construction and restoration continued with the support of local communities rather than the central state.

The inscriptions also often contain Buddhist poems that reveal the development of aesthetics and liturgical practices over time. While symbolic in nature, these poems can be used to trace the influence of particular schools, such as Chan (Zen) or Huayan, on the temple community. The language used also shows the evolution of linguistics and calligraphic script preferences from the Tang to the Qing dynasties. Monastic biographies such as the Xu Gaoseng Zhuan record the role of several Lingyan monks who were noted for their dedication to transmitting the Tripitaka teachings and establishing meditation centres. In this context, narrative sources provide personal data and the complex structure of monastic community life, including office hierarchies, duration of training, and intermonastic relations.

Administrative records in Jinan’s local archives show that the Lingyan Temple had a close relationship with the provincial government. Official letters about getting permission for renovations, assigning workers, and holding public ceremonies show how important the temple was to politics in the area. When making festival calendars, many documents show that abbots and local officials wrote to each other directly. The historiographic significance of these sources is enhanced through the triangulation of document types. For instance, older inscriptions can verify or amend data in the LYZ. In the meantime, biographical stories and administrative papers give a social context that is often missing from official or religious texts.

Along with the content, it’s also important to look at the physical condition of manuscripts and inscriptions. Natural causes, vandalism during the Cultural Revolution, and time have all damaged many primary sources in Lingyan. Therefore, conservation and digitisation efforts have become integral to modern historiographic studies. Collaboration with institutions such as Shandong University and the Chinese Academy of Social Sciences has become vital. In order to develop a positivistic approach, all of these data are codified through a thematic and spatial coding system. This means that each inscription or document is not only read as a single narrative, but is connected to the physical location in the temple complex, the time setting, and the relationships between the actors. This produces a relational map of historiography that allows for multi-layered interpretation.

The characteristics of primary bibliographic sources in empirically constructing the historical narrative of the Lingyan Temple, this section highlights the collection process, challenges, and analytical approaches used for relevant historical documents. The focus is on assessing each source’s credibility, internal consistency, and production context. One of the main challenges is the limited textual variants. The Lingyan Zhi (LYZ), the only available temple gazetteer, was compiled in 1696 by Li Xingzu during the Qing Dynasty after earlier versions were lost due to conflict. The absence of an earlier version makes it difficult to verify the narrative, especially since the LYZ shows several chronological inconsistencies. For example, the claim that Xuanzang translated the sutra in Lingyan in 629 CE contradicts the Xu Gaoseng Zhuan, which recorded Xuanzang as still in India. This highlights the importance of triangulating sources between textual narratives, epigraphic records, and material evidence.

Buddhist historical texts such as gazetteers (zhi 志) generally reflect local interests, including the glorification of regional figures and the construction of religious images that emphasise the spiritual prominence of a site. In the case of Lingyan Temple, the attempt to associate national figures such as Xuanzang and Lingrun with the site is more contextual and politico-religious than based on solid factual evidence. Furthermore, the redaction process during the compilation of the LYZ in the 17th century leaves potential errors in the placement of figures, events, and even temple structures. This creates biases that must be deconstructed using critical historical and contextual approaches.

## Discussion

Lingyan Temple’s position on the slopes of Mount Tai was not simply a topographical choice, but rather the result of a highly strategic cosmological-political configuration. Mount Tai, as the vertical axis in Chinese Liberation cosmology, symbolising the connection between heaven and earth, granted Lingyan ritual legitimacy through its connection to the imperial fengshan. The temple also served as a transit node between Jinan and the state’s ritual centre, making it both a sacred space and a centre for local diplomacy. Modern Geographies of Religion approaches view such places as symbolic constructions fraught with hierarchy, legitimacy, and politics (Obadia, 2025). In addition to its spatial and cosmological significance, the analytical methodology of this study adheres to a positivist verification framework. This methodological triangulation demonstrates the application of the positivist approach as an empirical testing mechanism, wherein textual, archaeological, and epigraphic evidence mutually corroborate one another, rather than relying solely on narrative interpretation.

While geography constructs spaces of legitimacy, Lingyan’s chronology reveals the dynamics of institutional adaptation to political change. Reconstructions based on epigraphy, archaeology, and historical documents reveal a recurring pattern of growth, decline, and regrowth, aligned with imperial and local patronage waves. This indicates that the temple’s existence was not always stable; rather, it pulsed with the needs for legitimacy and its adaptive capacity within political-imperial circles. This approach approaches a rhythmic, rather than linear, temporality increasingly relevant in studying institutional cycles (Sefer and Kingsford, 2016; Matsumoto, 2022). The textual and artefactual dimensions, temple bibliographies, add complexity to the spatial–temporal dimensions of legitimacy narratives. Lingyan Zhi, monk biographies, and diques infrastructure constitute an “architecture of memory” that fosters sacred imagery while concealing ideology. Through an archaeotextuality approach, texts are not read simply as narrative sources but are examined through correlation with inscriptions and physical structures. This historical correction opens up insights into how temple narratives were reconstructed to maintain a consistent identity. This concept aligns with how Buddhist epigraphy during the Northern Dynasties structured power through implicit language and space (Copplestone, 2024).

The study addresses the chronological inconsistencies in historical sources, including the disputed 446 CE destruction decree (Liu et al., 945/1975; Ma, 1998; Sengyou (519/1987), the dual attribution of Master Fading’s actions [as noted in *Gaoseng Zhuan* 高僧傳 (Huijiao, 1987) and *Xu Gaoseng Zhuan* 續高僧傳 (Daoxuan, 645/1988], and the anachronistic claims regarding Xuanzang’s translation. These discrepancies were re-evaluated through cross-source verification, transforming textual ambiguity into substantiated historical understanding. This process not only clarifies Lingyan Temple’s historical trajectory but also highlights how Buddhist historiography navigates temporal authority.

Beyond resolving textual contradictions, the study contributes to the geography of religion. It extends current frameworks by integrating the temporal and historiographical dimensions of sacred space. Building on Wu’s Regional Religious System (RRS), which links temples to political and pilgrimage networks, and Obadia’s *Geographies of Religion*, which examines the symbolic construction of sacred space, this study introduces the concept of “sacred space in motion.” This model views sacredness as a dynamic process shaped by political cycles of patronage, destruction, and renewal, rather than a static spatial arrangement. A comparative perspective situates Lingyan Temple within the broader East Asian Buddhist context. Similar patterns of geomantic alignment and cyclical reconstruction at Shentong and Langgong Temples, as well as in Korea’s Beopjusa and Japan’s Koyasan, demonstrate that Lingyan’s development reflects a regional phenomenon of “sacred space in motion.”

These three dimensions, geographic, chronological, and textual, create what can be called a sacred space in motion, where environment, time, and memory interpenetrate. Lingyan is not a dry monument but an institutional organism that pulsates in relation to the state, community, and landscape. The temple’s architecture reflects human-animal-political relationships through vertical orientation, pilgrimage circulation, and the layout of inscriptions—similar to findings in studies of global sacred space geography (RRS) that assess the interconnectedness of space and place with regional systems (Wu, 2022). Theoretically, this discussion expands the discourse on sacred space by emphasising that sacredness is not inherent but historically constructed through the interaction of material, time, and text. This study proposes a positivist model that combines spatial-historical analysis, chronological reconstruction, and philological criticism to produce an empirical and comparative historiography of the temple. This “space–time-text” model, although applied to Lingyan, is relevant for studies across Asia and the world, as a tool for reconstructing religious institutions in the context of modern sociopolitical and symbolic transformations.

## Conclusion

This study has demonstrated that the historiography of Lingyan Temple is best understood through the integrated lens of geography, chronology, and bibliography, revealing sacred space as a dynamic construct shaped by cosmological symbolism, cycles of imperial and local patronage, and layers of textual memory. The findings highlight that geography was not a passive backdrop but an active agent of legitimacy and resilience; chronology unfolded in adaptive cycles rather than linear progress; and bibliographic sources, when critically triangulated with epigraphic and archaeological evidence, served as ideological instruments and empirical data. Methodologically, the study advances a positivist framework that bridges material, temporal, and textual evidence, offering a transferable model for comparative religious heritage research. Nevertheless, limitations remain, notably the uneven survival of sources across periods and the interpretive challenges posed by interpolated gazetteers and fragmentary inscriptions. The “sacred space in motion” model presented here contributes to the development of spatial religion theories by embedding them within a historical and historiographical context, offering a conceptual framework for examining Buddhist heritage and religious landscapes across East Asia. Future research should build on the archaeotextual approach by incorporating digital epigraphy, historical spatial mapping, and comparative studies of East Asian temple networks. Such efforts will further refine our understanding of how sacred spaces are constructed, contested, and revitalized over time.

## Data Availability

The raw data supporting the conclusions of this article will be made available by the authors, without undue reservation.
